# Supplementary material for: Content-rich biological network constructed by mining PubMed abstracts
Source: BMC Bioinformatics. 2004 Oct 8;5:147. doi: 10.1186/1471-2105-5-147 (PMC528731; doi:10.1186/1471-2105-5-147)
Supplement: Additional File 2 — The original results of the above study (non-essential files are deleted to keep the file size under the limit set by BMC bioinformatics). [file 1471-2105-5-147-S2.bz2 › chilibotAdditionalFile2/dip10/265ID7997270E1066/html/MAPK1_ARAF1.html]

 


 **MAPK1** and **ARAF1** 
  
Found 67 abstracts in PubMed, retrieved 10.  
 

 What does Google say? 
 PDF only 
| .edu only 

---

**Interactive relationship** (e.g. stimulation, inhibition, etc)

**Neutral relationship**- Raf comprises a family of three kinases, A Raf  [ **ARAF1** ] , B Raf and Raf 1, which are best known as key regulators of the MEK MAPK  [ **MAPK1** ]  ERK  [ **MAPK1** ]  cascade.  Ref: 11950876 J Cell Sci, 2002

**Non-interactive relationship** (e.g. studied together, co-existance, homology, etc.)

- GLP1 stimulated activation of Erk  [ **MAPK1** ]  is blocked by inhibitors of MEK, but GLP1 does not induce the activation of A Raf  [ **ARAF1** ] , B Raf, C Raf, or Ras.  Ref: 12364324 J Biol Chem, 2002
- These studies suggest that the ability of Ras and BCR ABL to activate NF kB involves an uncharacterized pathway which does not involve classic IKK activity and which suppresses the TNF induced IKK pathway through a Raf  [ **ARAF1** ]  MEK Erk  [ **MAPK1** ]  independent mechanism.  Ref: 12851413 J Biol Chem< MedlineTA>J Biol Chem, 2002
- Thus, the suppression of integrin activation is a Raf  [ **ARAF1** ]  kinase induced regulatory event that can be mediated independently of bulk activation of the ERK  [ **MAPK1** ]  MAP kinase pathway.  Ref: 12134066 Mol Biol Cell, 2002
- To investigate the importance of Raf MEK ERK  [ **MAPK1** ]  signaling in human hematopoietic cell growth, the effects of three different Raf genes, A Raf  [ **ARAF1** ] , B Raf and Raf 1, on cell cycle progression and regulatory gene expression were examined in TF 1 cells transformed to grow in response to beta estradiol regulated DeltaRaf ER genes.  Ref: 12429936 Cell Cycle< MedlineTA>Cell Cycle, 2002
- Gastrin activated transcription through a Raf  [ **ARAF1** ] , Mek and Erk  [ **MAPK1** ]  dependent but Ras independent pathway.  Ref: 12891554 Gastroenterology, 2003
